# Solitary Fibrous Tumor Arising from the Sphenoid Sinus

**DOI:** 10.1155/2009/316042

**Published:** 2009-08-17

**Authors:** Kenji Takasaki, Takeshi Watanabe, Tomayoshi Hayashi, Naoe Kinoshita, Hidetaka Kumagami, Haruo Takahashi

**Affiliations:** ^1^Department of Otolaryngology Head and Neck Surgery, Nagasaki University Graduate School of Biomedical Sciences, Nagasaki 852-8501, Japan; ^2^Department of Pathology, Nagasaki University Hospital, Nagasaki 852-8501, Japan

## Abstract

Solitary fibrous tumor (SFT) is an uncommon neoplasm that usually arises from the pleura. To our knowledge, only 30 cases of SFTs in the nasal cavity and paranasal sinuses have been reported in the literature. We describe an SFT that arose from the right sphenoid sinus and extended to the nasal cavity and epipharynx. The tumor was completely removed by endoscopic sinus surgery without complication. The patient is taking an uneventful course without any evidence of recurrence of the disease 8 months after surgery now.

## 1. Introduction

Solitary fibrous tumor (SFT), also known as benign fibrous mesothelioma or submesothelial fibroma, is an uncommon neoplasm first described as a primary spindle cell tumor of the pleura in 1931 [1]. The majority of the tumors originate from the pleura, but SFTs can also be derived from other serosal membranes. Due to its mesenchymal origin, SFTs have been reported to originate in a wide variety of extrapleural locations such as the abdomen, extremities, and vulva [2]. Recently, SFTs in the nasal cavity and paranasal sinuses treated by endoscopic sinus surgery (ESS) have been reported [3–8], but there are only a few reports of an SFT in the sphenoid sinus treated by ESS. We describe an SFT that originated in the right sphenoid sinus and extended to the nasal cavity and epipharynx and was successfully treated by ESS.

## 2. Case Report

**Figure 1:**
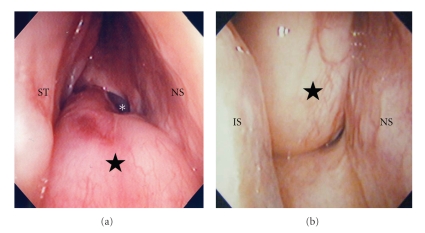
Endoscopic images of the tumor (★) in the right nasal cavity viewed from the superior (a) and inferior (b) directions. The base of the tumor is seen in the lateral wall of the ostium of the sphenoid sinus (∗). ST: superior turbinate, IT: inferior turbinate, NS: nasal septum.

The patient was a 74-year-old woman with a right nasal tumor that had caused progressive right nasal obstruction over two years. Nasal endoscopy revealed a pinkish tumor arising from the ostium of the right sphenoid sinus and extending to the right nasal cavity and epipharynx (Figures 1(a) and 1(b)). 

**Figure 2:**
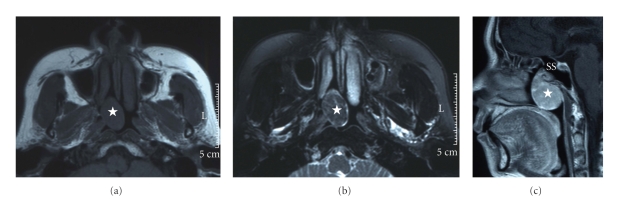
T1- (a) and T2-weighted (b) magnetic resonance (MR) images of the tumor (★). Gd-enhanced sagittal MR image (c) revealed that the tumor originated in the right sphenoid sinus (SS).

Plane-computed tomography (CT) showed a soft tissue density in the nasal cavity, sphenoid sinus, and nasopharynx without bone destruction on the right side. Magnetic resonance imaging (MRI) showed a mass with a hypointense signal on T1-weighted images (Figure 2(a)) and heterogeneous hypo-and hyperintense signals on T2-weighted images (Figure 2(b)). The mass showed prominent and inhomogeneous enhancement with gadolinium and was also revealed to be originated from the right sphenoid sinus (Figure 2(c)). 

**Figure 3:**
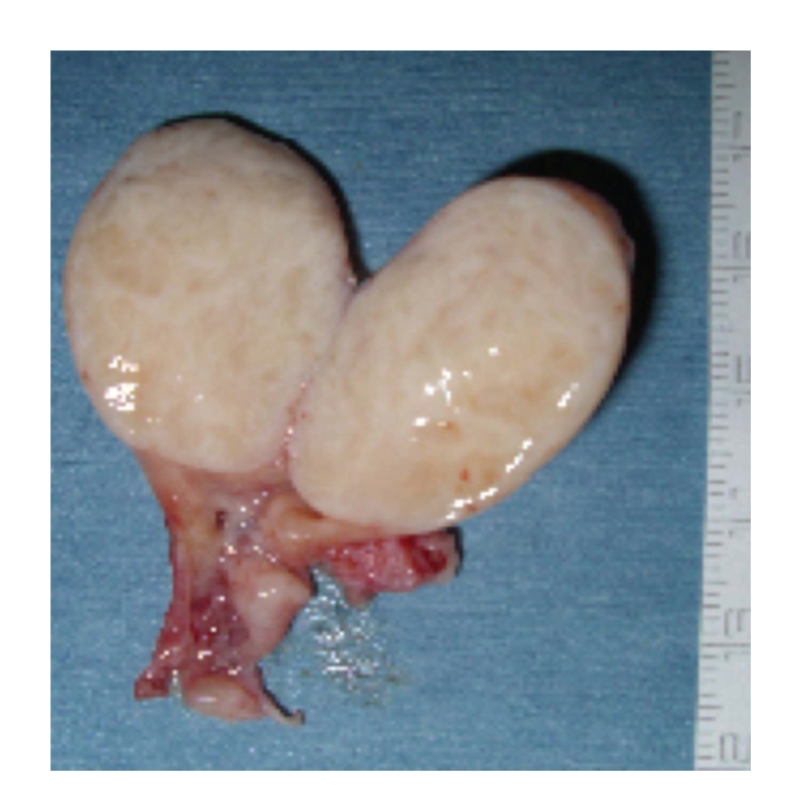
Macroscopic features of the excised tumor.

The ESS was performed under general anesthesia. During the sphenoethomoidectomy, the root of the tumor was clearly identified on the lateral edge of the ostium of the right sphenoid sinus. The tumor was successfully resected “en bloc” and was removed through the oral cavity (Figure 3). The postoperative course has been uneventful without signs of recurrence at eight months after surgery.

**Figure 4:**
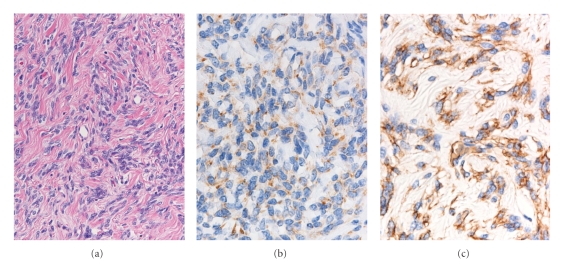
Histopathological features of the tumor. (a) the spindle cells are arranged in a patternless fashion in the collagenous matrix on hematoxylin-eosin staining (×200). (b) the vast majority of the tumor cells were stained diffusely and strongly for CD34 (×400). (c) cytoplasm in the numerous tumor cells expressed the Bcl-2 (×400).

Histopathological examination of the tumor revealed the spindle cells showing a patternless arrangement within the collagenous matrix and numerous thick-walled vessels with dilated vascular spaces (Figure 4(a)). Immunohistochemically, the tumor cells were stained positively for CD34 (Figure 4(b)) and Bcl-2 (Figure 4(c)), but were not stained for S-100 proteinor c-kit; thus, a diagnosis of SFT was established.

## 3. Discussion

The first identification of SFT as a distinct entity is generally credited to Klemperer and Rabin [1]. Plueral SFTs have long been supposed to originate from the surface mesothelium of the pleura. Today, however, most authors agree that they originate from submesothelial connective tissue. This hypothesis is based on immunohistochemical and ultrastractural findings [9–12] as well as on the occasional occurrence of this tumor in extrapleural sites. Histologically, SFTs are formed by plump spindle cells arranged in a patternless fashion in a collagenous background. Typically, there are hyper-and hypocellular areas and prominent vascularity within the lesion that result in a hemangiopericytoma-like pattern. Recently, CD34, a transmembrane glycoprotein found on the surface of hematopoietic progenitor cells, has been considered a positive marker for SFT. Although a varying degree of CD34 immunoreactivity is observed in the neurofibroma and schwannoma, they are usually strongly positive also for S-100 protein, in contrast to SFT [11].

An SFT typically presents as a slow-growing, painless mass. To date, 30 cases of SFTs in the nasal cavity and paranasal sinuses have been reported in the English literature [3–8, 13–19]. Most of these patients complained of a unilateral intranasal mass, nasal obstruction, rhinorrhea, and epistaxis. Other signs and symptoms, such as exophthalmos, epiphora, anosmia, headache, facial pain, and visual disturbances, may also be present. In some cases, SFTs showed bone destruction and defect on CT, but were usually smoothly surfaced and encapsulated. In all cases of SFT, including the present case, MRI showed iso-hypointensity on T1-weighted images and enhancement with gadolinium contrast. T2-weighted images varied from low intensity to high intensity [13].

SFTs have, however, been reported to be malignant in 13%–23% of cases. Factors associated with malignancy include high cellularity, more than 4 mitoses per 10 high-power fields, pleomorphism, hemorrhage, and necrosis . The diagnosis of malignancy is based on both clinical features and these histologic findings [2, 12, 15]. Prognostic factors are not clearly defined for SFTs in the head and neck region. Factors that predispose to local recurrence in nonhead and neck SFTs are a tumor diameter larger than 10 cm, the presence of a malignant component to the histologic findings, and microscopically positive surgical margins [20]. The study by Gold et al. [20] also showed that positive margins correlated with a large tumor. As might be expected in the nasal cavity and paranasal sinuses, all tumors were smaller than 10 cm in diameter in head and neck SFTs. In contrast to tumors found in other anatomic locations, such as the trunk and lower extremities, tumors of the head and neck lesions present a unique anatomic challenge by virtue of their relationship to adjoining structures. Therefore, this high rate of positive margins most likely reflects the tumor location rather than its biological features. In the previous literature, two patients were reported as local recurrence [15, 16], but these patients were controlled by wide complete excision. Patients with positive margins should therefore be closely followed up for several years.

Since ESS for SFT was first described in 2003 [3], SFTs in the nasal cavity and paranasal sinuses have been treated successfully by ESS [3–8] in the cases that SFT was limited in the nasal cavity and paranasal sinuses, if there is no invasion of the anterior cranial fossa and no extension to the infratemporal fossa. In the present case, we could successfully treat the SFT in the sphenoid sinus by ESS, because the anatomical features of the sphenoid sinus and its surrounding structures in terms of their relevancy for performing an endoscopic sphenoidotomy were already clarified [21]. 

ESS is considered the first-line approach for achieving the complete removal of benign tumors such as an SFT, as long as it is delineated within the level of the nasal cavity and paranasal sinuses except the frontal sinus. However, the STFs extended to the skull base; these tumors should be removed through a transcranial approach or an open transfacial approach [13–19].
